# Sleep in *Kcna2 *knockout mice

**DOI:** 10.1186/1741-7007-5-42

**Published:** 2007-10-09

**Authors:** Christopher L Douglas, Vladyslav Vyazovskiy, Teresa Southard, Shing-Yan Chiu, Albee Messing, Giulio Tononi, Chiara Cirelli

**Affiliations:** 1 Department of Psychiatry, University of Wisconsin, Madison, WI, 53719, USA; 2 Waisman Center, University of Wisconsin, Madison, WI, 53719, USA; 3 Department of Physiology, University of Wisconsin, Madison, WI, 53719, USA

## Abstract

**Background:**

*Shaker *codes for a *Drosophila *voltage-dependent potassium channel. Flies carrying *Shaker *null or hypomorphic mutations sleep 3–4 h/day instead of 8–14 h/day as their wild-type siblings do. Shaker-like channels are conserved across species but it is unknown whether they affect sleep in mammals. To address this issue, we studied sleep in *Kcna2 *knockout (KO) mice. *Kcna2 *codes for Kv1.2, the alpha subunit of a Shaker-like voltage-dependent potassium channel with high expression in the mammalian thalamocortical system.

**Results:**

Continuous (24 h) electroencephalograph (EEG), electromyogram (EMG), and video recordings were used to measure sleep and waking in *Kcna2 *KO, heterozygous (HZ) and wild-type (WT) pups (P17) and HZ and WT adult mice (P67). Sleep stages were scored visually based on 4-s epochs. EEG power spectra (0–20 Hz) were calculated on consecutive 4-s epochs. KO pups die by P28 due to generalized seizures. At P17 seizures are either absent or very rare in KO pups (< 1% of the 24-h recording time), and abnormal EEG activity is only present during the seizure. KO pups have significantly less non-rapid eye movement (NREM) sleep (-23%) and significantly more waking (+21%) than HZ and WT siblings with no change in rapid eye movement (REM) sleep time. The decrease in NREM sleep is due to an increase in the number of waking episodes, with no change in number or duration of sleep episodes. Sleep patterns, daily amounts of sleep and waking, and the response to 6 h sleep deprivation are similar in HZ and WT adult mice.

**Conclusion:**

Kv1.2, a mammalian homologue of Shaker, regulates neuronal excitability and affects NREM sleep.

## Background

By screening for mutations that affect daily sleep amount in fruit flies we have recently demonstrated that the *Drosophila *gene *Shaker *plays a major role in controlling sleep amount [1]. Flies carrying the null allele *minisleep *(*Shaker*^*mns*^), or several other null alleles of *Shaker, *sleep only 3–4 h/day, while their wild-type controls sleep 8–14 h/day [1]. Similar to their controls, however, *Shaker *mutant flies mostly sleep at night, even when kept in constant darkness, and show a sleep rebound after sleep deprivation, suggesting that they have a normal circadian and homeostatic regulation of sleep [1]. The *Shaker *locus encodes the alpha (pore-forming) subunit of a tetrameric potassium channel that passes a voltage-activated fast-inactivating current, I_A_. In *Drosophila, *I_A _plays a major role in the control of membrane repolarization and transmitter release [2]. *Hyperkinetic *codes for the beta (regulatory) subunit of the Shaker channel, which positively modulates I_A _[3]. Recently, we found that *Hyperkinetic *loss of function mutations also result in reduced sleep [4]. Thus, in *Drosophila*, two different genes affecting the Shaker-related current have a strong effect on sleep.

While there is only one *Shaker *gene in *Drosophila*, at least 16 genes coding for alpha subunits of voltage-dependent potassium channels are present in mammals [5,6]. Based on sequence similarity, the closest mammalian homologues of the *Drosophila *Shaker are the alpha subunits of the Kv1 family, while the Kv2, Kv3, and Kv4 families are more distantly related ("Shaker-like"). Kv1 channels activate in the subthreshold voltage range in many cell types, and can act as extremely diverse regulators of neuronal excitability. In the supragranular layers of the rat cerebral cortex, for instance, most pyramidal cells contain different combinations of Kv1.1, Kv1.2, Kv1.3, and Kv1.4 subunits, localized in both the somatodendritic and the axonal compartments [7]. Most Kv1 subunits produce a slowly-inactivating current, originally defined as "D current" in the hippocampus [8]. However, depending on the subunit composition, the presence of beta (modulatory) subunits, and their anatomical position, the exact biophysical properties and functional properties of the Kv1-mediated currents can vary [7,9-11].

It is presently unclear whether potassium channels containing any of the eight members of the Kv1 family of alpha subunits can affect mammalian sleep as powerfully as Shaker affects *Drosophila *sleep. Here, we studied the potential role of Kv1.2 by measuring sleep and the response to sleep deprivation in mice carrying a null mutation of *Kcna2*, the murine gene coding for Kv1.2. We focused on this subunit because it is, together with Kv1.1 and Kv1.4, the most highly expressed in the rodent brain. Specifically, Kv1.2 is prominently and homogenously expressed in the rat thalamocortical system [12-14], which is important for the generation of sleep rhythms [15]. Moreover, its expression is modulated by synaptic activity [14].

Some of these findings have previously been presented in abstract form [16].

## Results

**Figure 1:**
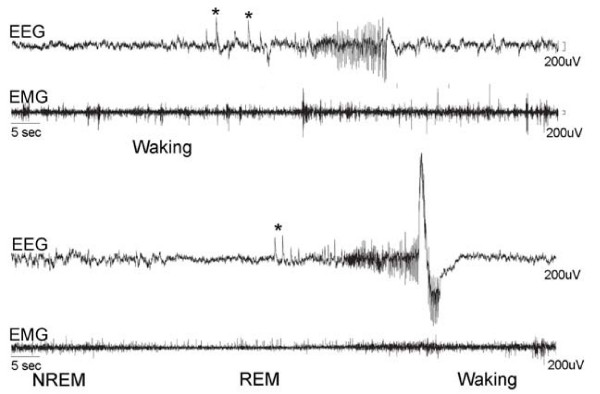
**Representative electroencephalographic (EEG) and electromyographic (EMG) recordings of non-fatal episodes of seizure occurring in two *Kcna2 *null pups during waking (upper two traces) and during REM sleep (lower two traces)**. The EEG shows some higher voltage, intermittent spikes (asterisks) followed by synchronized activity (9–10 Hz) with high-voltage spikes.

We found that knockout (KO) pups appeared healthy and grew normally during the first 2 weeks of life. At P16, however, KO pups were smaller than their littermates (-14% vs wild-type (WT), p = 0.032; -11% vs heterozygous (HZ), p = 0.047). KO pups also did not show clear differences from their littermates in their ability to recover from surgery and anesthesia. Specifically, the required dose of anesthetic and wake-up time (time to regain purposive motility after anesthesia) were similar in all pups. Similarly, post-operative general appearance and changes in weight did not differ among pups. Previous observations had indicated that all KO pups die by P28, with an average lifespan of 17 days [17]. By using continuous electroencephalograph (EEG) and video recordings from P16 to P19 we confirmed the conclusion by Brew et al, which was based on behavioral observation, that the cause of death was an isolated episode of generalized seizure [17]. Seizures were never seen, either at the behavioral or the EEG level, in HZ mice, and occurred rarely in KO pups before the terminal episode. Specifically, at P17, the day that was used for the sleep analysis described below, four KO pups did not have seizures, while the other three pups had an average of 3.3 seizures (± 0.6), accounting for 0.18% (± 0.12) of the their total recording time (2.5 min/24 h). Most seizures we recorded between P16 and P18 occurred during waking (47%) or during rapid eye movement (REM) sleep (30%), as shown in Figure 1. Obvious seizure behavior was always associated with an ictal EEG pattern, and abnormal spiking EEG activity was only recorded in KO pups during overt generalized seizure behavior, which is described in detail in another study [17]. One of the seven KO pups died following its first recorded seizure, one after its second seizure, and one after its third seizure. In one KO pup that was EEG recorded through P20 we did not detect any seizure. Sixteen other KO pups were implanted at P16 but died of seizures before we could collect 24 h of EEG recording. Based on direct visual observation and by reviewing the 24-h video recordings, we could not identify clear signs of behavioral hyperactivity in KO pups. In fact, until the occurrence of the first seizure, KO pups could not be distinguished from their littermates based on their general behavior and locomotor activity levels. Moreover, the 24-h integrated electromyogram (EMG) activity during waking, expressed as a percentage of the 24-h integrated EMG activity during non-rapid eye movement (NREM) sleep, was similar in KO pups relative to their HZ and WT siblings (mean ± SEM, WT = 325.7% ± 29.8; HZ = 363.1% ± 77.6; KO = 280.7 ± 91.4, unpaired *t *test; see Methods for details).

**Figure 2:**
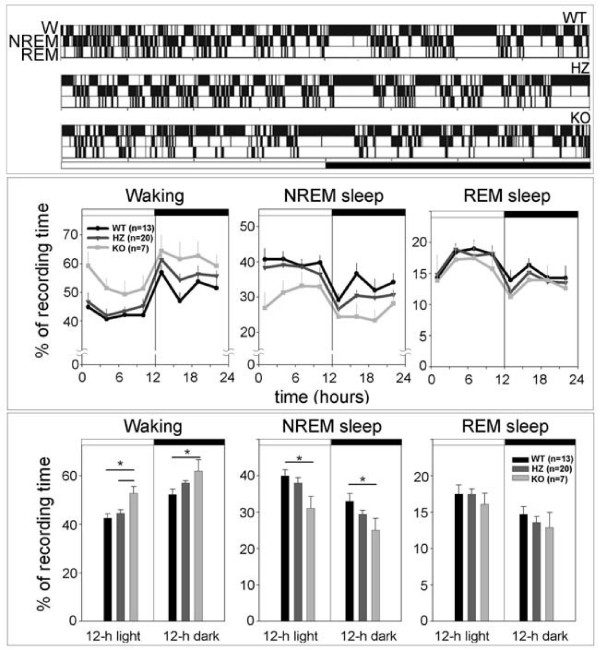
**Hypnograms and sleep and waking patterns**. Upper panel: representative 24-h hypnograms from a *Kcna2 *null (KO), heterozygous (HZ), and wild-type (WT) pup at P17. White and black bars indicate the light and dark period, respectively. W, waking; NREM, NREM sleep; REM, REM sleep. Middle and lower panels: 24-h sleep and waking patterns and 12-h sleep and waking amounts (mean ± SEM) in *Kcna2 *KO, HZ, and WT pups at P17. *, p < 0.05 (Tukey's test).

**Figure 3:**
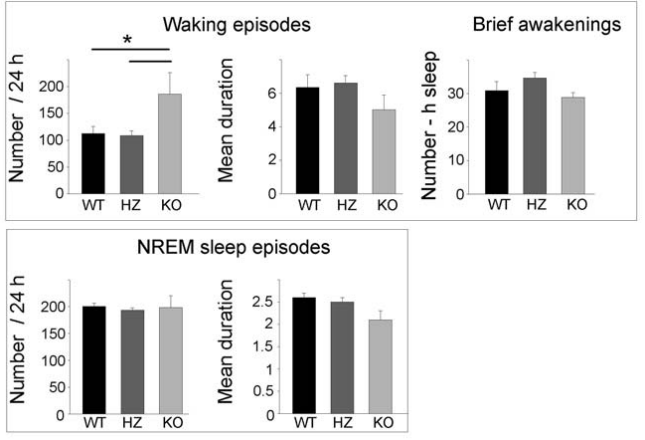
**Number and duration (in min) of waking and NREM sleep episodes in *Kcna2 *KO, HZ, and WT pups at P17**. Brief awakenings were defined as uninterrupted waking episodes shorter than 16 s and were computed per hour of total sleep *, p < 0.05 (Tukey's test).

**Figure 4:**
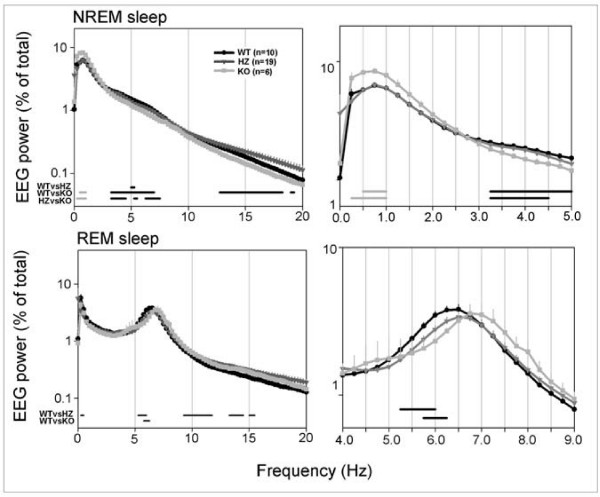
**Left panels: 24-h NREM and REM sleep EEG power density spectra (0–20 Hz) in *Kcna2 *KO, HZ, and WT pups at P17**. The right panels show at higher magnification some of the frequency bands indicated on the left. The EEG power spectrum for each 0.5 Hz frequency bin is expressed as a percentage of the total (0–20 Hz) EEG power. Grey and black bars indicate differences between genotypes (p < 0.05, unpaired *t *test on log-transformed spectral values). Grey and black bars indicate frequency bins where EEG power in KO pups was highest and lowest among genotypes, respectively.

As shown in Figure 2, at P17 all pups showed consolidated periods of sleep and waking, a clear diurnal rhythmicity (sleep prevailed during the light period), and robust entrainment to the light-dark cycle. KO pups, however, spent significantly less time in NREM sleep (-23%; p < 0.001) and significantly more time in waking (+21%; p < 0.001) than WT pups, with a 10% (non-significant) decrease in REM sleep time. The differences between KO and WT mice were significant also when the time spent in waking and NREM sleep was computed separately for the light and the dark phase (Figure 2, lower panel). During the light period, KO pups also spent more time in waking (+13%; p < 0.01) than HZ pups, while waking, NREM, and REM sleep amounts in HZ pups did not differ from those in WT pups. The increase in waking time in KO pups was mainly due to an increase in the number of waking episodes, while their duration, as well as the number of brief awakenings, did not differ among genotypes (Figure 3). NREM sleep episodes did not differ in their number across genotypes, while their duration showed a tendency toward a decrease in KO pups (Figure 3). Subtle differences were present among genotypes in the EEG power spectrum during NREM and REM sleep. During NREM sleep, KO mice showed a higher EEG power in the low-frequency range (0.5–1.0 Hz), and a lower EEG power in the 3.5–7 Hz frequency range relative to both HZ and WT pups (Figure 4). Two KO pups were also recorded at P19, and the results were similar to those seen at P17. Specifically, in these two animals, waking time/24 h increased by 19% and 15% relative to WT and HZ littermates, respectively, while NREM sleep/24 h decreased by 30% and 28% (WT, n = 11; HZ, n = 20; p < 0.05).

**Figure 5:**
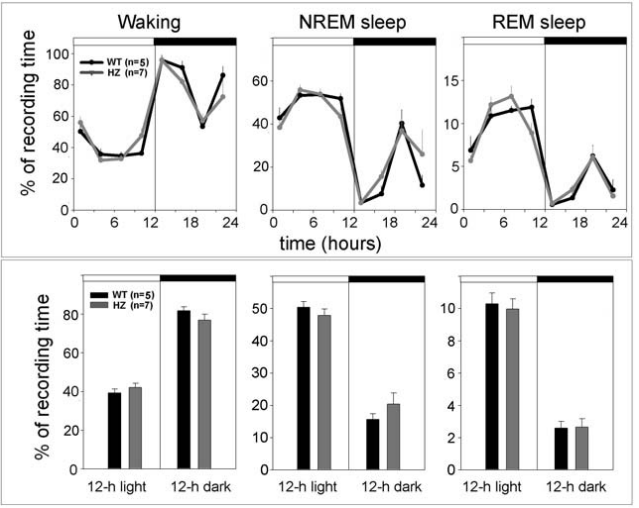
**The 24-h sleep and waking patterns (upper panel) and 12-h sleep and waking amounts (lower panel) in *Kcna2 *adult HZ and WT mice**. White and black bars indicate the light and dark period, respectively. Values are mean ± SEM.

**Figure 6:**
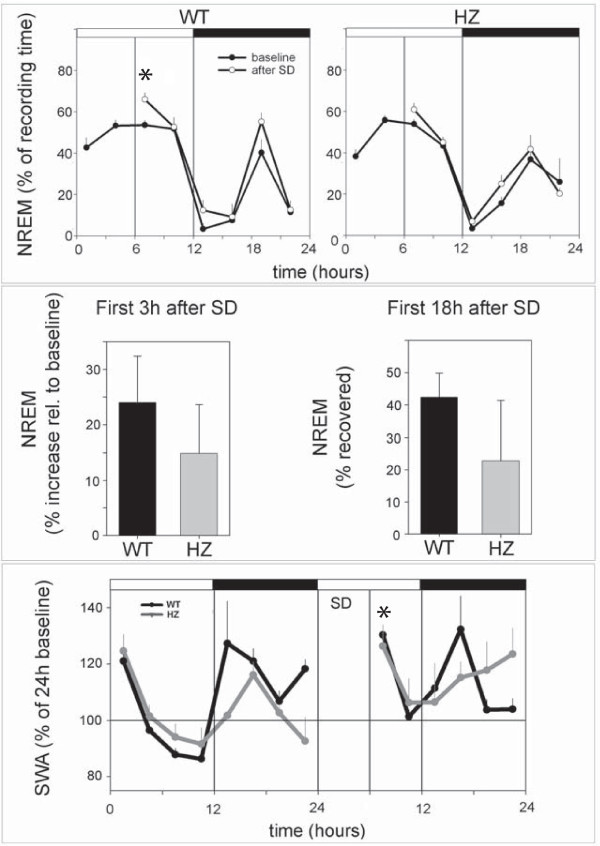
**Changes in NREM sleep duration and slow wave activity (SWA, 0.5–4.0 Hz) following 6 h of sleep deprivation (SD, during the first half of the light period) in *Kcna2 *adult HZ and WT mice**. The asterisk in the upper WT panel indicates a significant increase in NREM sleep duration during the first 3 post-SD sleep relative to baseline (p < 0.05, paired *t *test). Middle panel, right: the amount of NREM sleep during the first 18 h after SD is expressed as a percentage of what was lost during the 6 h of SD. Lower panel: the asterisk indicates a significant increase in SWA during the first 3 h post-SD sleep relative to the corresponding baseline interval (p < 0.05, paired *t *test, both WT and HZ). All values are mean ± SEM.

Adult (P67) HZ and WT mice did not differ in the amount of time spent in waking, NREM, and REM sleep (Figure 5), nor in any other sleep parameter examined, including the number and duration of waking episodes, the number and duration of NREM episodes, and the number of brief awakenings (data not shown). The EEG power spectrum in waking, NREM and REM sleep during baseline was also similar in the two groups (data not shown). During the first 3 h of recovery sleep following sleep deprivation, both HZ and WT mice spent more time in NREM sleep than during baseline, although the increase reached significance only in the WT group (Figure 6, upper panel). When expressed as percentage increase in NREM sleep time across the first 3 h of recovery sleep, this sleep rebound did not differ between HZ and WT mice (Figure 6, middle panel, left). The total amount of NREM sleep recovered during the first 18 h after sleep deprivation was also similar in HZ and WT mice (Figure 6, middle panel, right). Finally, during the first 3 h of recovery slow wave activity (SWA, 0.5–4.0 Hz) during NREM sleep increased significantly, relative to baseline, in both HZ and WT mice (Figure 6, lower panel). The overall SWA time course did not differ between the two groups before or after sleep deprivation (Figure 6, lower panel).

## Discussion

In this study we confirmed that *Kcna2 *null mice, in which the expression of the voltage-dependent potassium channel alpha subunit Kv1.2 is completely abolished, die within the first 3–4 weeks because of an episode of generalized seizures [17]. Seizures and a reduced lifespan have also been described in *Kcna1 *null mice, in which the expression of the Kv1.1 subunit is abolished [18], as well as in *Kcnab2 *null mice [19], lacking the modulatory subunit beta 2, which co-distributes extensively with Kv1.1 and Kv1.2 in the adult rat brain [11]. About 50% of *Kcna1 *null mice die suddenly between the third and the fifth week of life, in many cases after an episode of generalized seizure, and the mice that survive to adulthood display frequent (1–2/h) spontaneous seizures throughout adult life [18]. The seizure behavior in the *Kcna2 *mice examined in this study appears even more severe than in *Kcna1 *null mice, because none of our KO pups survived to adulthood. Thus, if seizure susceptibility provides any indication of the role of different Kv1 subunits in the control of neuronal excitability, Kv1.2 appears to have a very important one. So far, neuronal excitability in *Kcna2 *null mice has been directly measured only in the medial nucleus of the trapezoid body, where it is decreased [17]. Thus, the brain regions responsible for the increased seizure susceptibility of *Kcna2 *null mice remain to be identified. We recently performed microinjections of an antibody against the extracellular portion of Kv1.2 in the cerebral cortex of adult rats. This antibody has been previously shown in vitro to block the Kv1.2-mediated current by up to 70% [20]. We found that while low doses were able to block EEG signs of slow wave sleep, high doses also produced seizures [21], suggesting that an increase in neuronal excitability in the cerebral cortex, due to a block of the Kv1.2-mediated current, is sufficient to trigger seizure activity.

Because of the reduced lifespan of our KO mice, we were forced to analyze their sleep pattern at P17. At this development stage the sleep-waking cycle shows a clear diurnal rhythm, is fully entrained to the light-dark cycle, and EEG signs of waking, NREM sleep, and REM sleep are fully developed [22]. Moreover, at P17 mouse pups can be separated from their mothers and continuously recorded for several days. We found that KO pups slept significantly less than HZ and WT pups, mainly because they spent less time in NREM sleep during both the light and the dark period. During our 24-h recordings seizure activity, at the behavioral and EEG level, was either completely absent (in four KO pups), or very rare (in three KO pups), accounting for less than 1% of the total recording time. Even when present, seizures seldom originated during NREM sleep. Moreover, the increase in waking time and the decrease in NREM sleep time were not more pronounced in the three KO pups that showed seizures relative to the other four (in fact, the two pups with the biggest changes in waking and sleep time had no seizures at P17). Thus, the decrease in NREM sleep time in KO pups cannot be due to the repeated occurrence of seizures originating during this phase of sleep. Furthermore, although we cannot completely rule out this possibility due to the low number of animals, it is unlikely that the presence of seizures at P17 could account for the observed changes in the sleep/waking cycle.

A role for some voltage-dependent potassium channels in the regulation of sleep has been suggested by previous studies, which focused on the Kv3 family. Mice lacking the potassium channel subunit Kv.3.1, which is expressed in fast-spiking, parvalbumin-containing interneurons in cerebral cortex, hippocampus, striatum, and the thalamic reticular nucleus, show a 20–50% decrease in the EEG power in the slow wave activity (SWA) range (2–3 Hz) [23]. However, the effect on this frequency band is seen in waking as well as in sleep, and thus its functional significance in relation to NREM sleep and its homeostatic regulation remains unclear [23]. Mice lacking the potassium channel subunit Kv.3.2, which is expressed in thalamocortical neurons and fast spiking GABAergic interneurons of the neocortex and hippocampus, do not differ from WT controls in daily sleep amount and in their response to sleep deprivation, but show some differences in the EEG power spectrum during NREM and REM sleep [24]. Finally, mice lacking both the Kv3.1 and the Kv3.3 subunit (Kv3.3 is expressed in most Kv3.1-expressing brain regions) show a 40% decrease in sleep amount during the light period and a 22% decrease during the dark period relative to their WT controls [23]. In contrast to what we found in Kv1.2-deficient pups, the decrease in sleep in these double KO mice is mainly due to a decrease in the duration of NREM episodes. Moreover, Kv3.1/Kv3.3 deficient mice are hyperactive, with an increase in both ambulatory and stereotypic activity, display frequent (every few seconds) spontaneous myoclonic jerks, and their hyperactivity correlates, across different genotypes, with the decrease in NREM sleep [23]. These results suggest either that the increased motor drive in Kv3.1/Kv3.3 KO mice causes sleep loss, or that both phenotypes result from the increased neuronal excitability of a single or multiple anatomical loci [23]. In Kv1.2-deficient pups, instead, the decrease in NREM sleep amount was not associated with signs of sleep fragmentation, because there were no differences in the number and duration of NREM sleep episodes, or in the number of brief awakenings, between KO, HZ, and WT mice. Interestingly, *Shaker *and *Hyperkinetic *short-sleeping flies also do not show signs of sleep fragmentation [1,25]. Sleep loss in Kv1.2-deficient pups was also not associated with hyperactivity, as documented by continuous video recordings and by measuring integrated EMG activity. Thus, the reduced amount of NREM sleep in our pups cannot be secondary to increased motor activity. Moreover, the brain regions responsible for the sleep phenotype of Kv1.2-deficient mice most likely are not involved in the control of motor activity.

*Shaker *and *Hyperkinetic *short-sleeping flies show a normal response to sleep deprivation [1,25], suggesting that Shaker-related currents do not regulate daily sleep amount by affecting the homeostatic regulation of sleep. Due to their age, we could not assess the response to sleep deprivation in Kv1.2-deficient pups. Thus, we do not know whether Kv1.2-related currents also affect sleep duration without affecting sleep homeostasis. The mechanism by which *Shaker *mutations regulate daily sleep amount in flies remains unknown, as it is the case for *Kcna2 *mutations in mice. One possibility is that, by affecting an ion channel that controls membrane repolarization, Shaker channels and their mammalian homologues could be close to the core cellular mechanisms of sleep. As mentioned before, in adult rats, intracortical injections of an anti-Kv1.2 antibody abolish or reduce EEG signs of NREM sleep on the injected cerebral cortex for up to 12 h after the injection. The effect is dose-dependent, reversible, and site-specific [21]. Thus, the pharmacological block of the Kv1.2-mediated current seems to be sufficient in vivo to maintain a long-lasting depolarization of cortical cells incompatible with the occurrence of NREM sleep. The current study in *Kcna2 *KO mice suggests, however, that such block must be almost complete for NREM sleep to be affected, because HZ mice, which express approximately half of the wild-type amount of Kv1.2, slept normally. This is also true in *Drosophila*, where only homozygous, but not heterozygous *Shaker *mutants are short sleeping [1]. Whether Kv1.2-mediated currents also affect human sleep is unknown. It is not known, for instance, whether human extreme short sleepers carry null or hypomorphic mutations in voltage-dependent potassium channels the same way *Shaker *short sleeping flies do. Intriguingly, however, a recent study identified auto-antibodies against members of the Kv1 family, including Kv1.2, in the brain of a patient affected by severe insomnia associated with Morvan's syndrome [26]. The insomnia improved after plasma exchange, suggesting that the presence of these autoantibodies was at least partially responsible for the loss of sleep.

## Conclusion

Kv1.2, a mammalian homologue of Shaker, regulates neuronal excitability and affects NREM sleep.

## Methods

*Kcna2*^-/- ^(KO) mice were generated as described in Brew et al [17]. KO, *Kcna2*^+/- ^(HZ) and *Kcna2*^+/+ ^(WT) mice (congenic in the *C57BL/6 b*ackground, > 23 generations) were born according to Mendelian proportions, indicating that the *Kcna2 *null mutation does not cause embryonic lethality. KO mice express no Kv1.2 mRNA or protein, while HZ mice express approximately half of the wild-type amount [17]. No changes in the expression of Kv1.1 and 1.6 mRNA have been observed in KO mice [17]. HZ mice are healthy, grow to adulthood, and are capable of breeding. KO mice, instead, appear healthy and develop normally during the first 2 weeks, but die suddenly between ~P12 and ~P28 due to an episode of generalized seizure, followed by full tonic extension, which in mice often results in fatal apnea [17]. Seizures were never observed at the behavioral or EEG level in HZ or WT mice, both of which had normal lifespan. As a distinct pattern of sleep and waking develops in neonatal mice only at the end of the second postnatal week [22], we implanted pups (n = 86) for sleep recordings at P16, when they were also old enough to survive in isolation. To reduce the stress of the implant procedure, pups were handled for at least 10 min twice per day while remaining with the dam and siblings for the 2 days before surgery (at P14 and P15). Pups were anesthetized with isoflurane (~1.4% in 100% O_2_) while an automated heating pad maintained body temperature. Small craniotomies were made with a sterile 26 ga needle above right and left parietal cortices (~1 mm lateral to the midline and ~1 mm caudal to the bregma suture) and one above cerebellum (midline, ~1 mm caudal to lambda). Wire electrodes for recording the EEG were created by placing a 1 mm 90° bend at the end of a 36 gauge Teflon-coated stainless steel wire. EEG electrodes were inserted through the craniotomies and fixed in place between the skull and the brain with dental cement. Two vinyl-coated braided stainless steel wire electrodes were placed in nuchal muscles for recording the EMG. Immediately upon recovery from anesthesia (within 15 min of the end of surgery pups were able to walk and eat), pups were housed individually in sound-proof, environmentally controlled recording chambers (12:12 LD, lights on 10:00 am; 30°C ± 1°C, food and water ad libitum). P16 pups are able to eat solid food and groom themselves. However, to ensure that the pups were adequately nourished, liquid rodent diet (F1268SP, Bio-Serv, Frenchtown, NJ) was also provided in addition to solid food.

Adult mice were anesthetized at P60 with isoflurane (~1.4% in 100% O_2_). Two gold plated miniature screw electrodes (0.9 mm diameter) were placed over the right and left occipital cortices (2 mm posterior to bregma, 2–3 mm lateral to the midline) and one over cerebellum (1 mm posterior to lambda, midline). Screw electrodes were wrapped with Teflon-coated stainless steel wire. Two vinyl-coated braided stainless steel wire electrodes were placed in nuchal muscles for recording of EMG. EEG and EMG electrodes were soldered to a flexible cable and the headcap assembly was affixed to the head using dental cement. Following surgery, adult mice were housed individually in sound-proof, environmentally controlled recording chambers (12:12 LD, lights on 10:00 am, 25°C ± 1°C, food and water ad libitum).

For both pups and adults, all electrodes were gathered into a flexible cable and connected via 24-channel commutators (Airflyte, Bayonne, NJ, USA) to a Grass Model 8 polygraph (Astro-Med. Inc., West Warwick, RI, USA). The EEG and EMG signals were amplified, conditioned by analog filters (EEG high-pass filter: -3 dB at 0.1 Hz; low-pass filter: -3 dB at 35 Hz; less than  – 35 dB at 128 Hz, EMG high-pass filter: -3 dB at 15 Hz; low-pass filter: -3 dB at 70 Hz; less than -35 dB at 128 Hz), and stored with a resolution of 128 Hz. The EEG and EMG signals were continuously recorded for 1–2 weeks using Sleep Sign™ software (Kissei Comtec America, Inc., Irvine, CA, USA). The 24-h polygraphic recordings were scored visually in 4-s epochs to identify behavioral states. EEG power spectra of consecutive 4-s epochs (Fast Fourier Transform routine, Hanning window) were calculated for the parietal-cerebellar derivations within the frequency range of 0–20 Hz. Continuous individual digital video recordings using infrared cameras were used to visually verify behavioral states. For EMG analysis, the total EMG power between 0 and 20 Hz was computed, and the average 24-h waking EMG value for each mouse was expressed as a percentage of its average 24-h NREM sleep EMG value. This frequency band was selected because we found that it roughly reflects the levels of locomotor activity, and in most cases can distinguish sleep from quiet waking and active waking. Sleep deprivation was performed at P68. For the first 6 h of the light period, mice were kept awake by introducing novel objects (i.e. nesting material, paper, wood blocks) and by tapping on the cage whenever the animals appeared drowsy or when slow waves appeared in the EEG. Mice were never disturbed when eating, drinking, grooming, or spontaneously exploring. The animals were left undisturbed with EEG, EMG, and video recorded for 18 h following the sleep deprivation.

Tail samples for genotyping were taken from each mouse at the conclusion of the study, after animals were killed. Sixteen implanted pups died between P16 and P17 before we could collect 24 h of EEG recording (genotyping showed that they were all KO). Overall, among the 86 implanted pups, there were 20 WT, 43 HZ, and 23 KO animals). Seven WT pups were excluded from the analysis because of poor quality of the EEG signal. The number of HZ was capped at 20, i.e. only the first 20 HZ pups with good EEG recordings were fully scored and used for the analysis. Animal protocols followed the National Institutes of Health *Guide for the Care and Use of Laboratory Animals *and were in accordance with institutional guidelines.

The weight of mouse pups was analyzed by descriptive statistics and *t *test (Statistica software, StatSoft, Inc., Tulsa, OK, USA). All arousal state-related data were analyzed by descriptive statistics and multifactorial analysis of variance (ANOVA), *t *test and post-hoc Tukey's test. One-way ANOVA and post-hoc Tukey's test were used to analyze vigilance state separately for the light and dark periods. A p value of less than 0.05 was considered significant. EEG scoring was done blind to genotype.

## Abbreviations

EEG, electroencephalogram

EMG, electromyogram

FFT, Fast Fourier Transform

HZ, heterozygous

KO, knockout

NREM sleep, non-rapid eye movement sleep

REM sleep, rapid eye movement sleep

SWA, slow wave activity

WT, wild-type

## Competing interests

The author(s) declares that there are no competing interests.

## Authors' contributions

CLD carried out surgery and polygraphic recordings, EEG analysis, and helped to draft the manuscript; VV performed EEG and power spectrum analysis, TS performed surgery and helped with EEG analysis; AM and SYC produced the mice and contributed to the behavioral analysis; GT designed the experiments, coordinated the development of the study, provided the economical support to the project, and helped in the last version of the manuscript; CC designed the experiments, coordinated and supervised the project, and wrote most of the manuscript. All authors read and approved the final manuscript.

## References

[B1] Cirelli C, Bushey D, Hill S, Huber R, Kreber R, Ganetzky B, Tononi G (2005). Reduced sleep in *Drosophila Shaker *mutants. Nature.

[B2] Schwarz TL, Tempel BL, Papazian DM, Jan YN, Jan LY (1988). Multiple potassium-channel components are produced by alternative splicing at the *Shaker *locus in *Drosophila*. Nature.

[B3] Chouinard SW, Wilson GF, Schlimgen AK, Ganetzky B (1995). A potassium channel beta subunit related to the aldo-keto reductase superfamily is encoded by the *Drosophila *hyperkinetic locus. Proc Natl Acad Sci USA.

[B4] Bushey D, Huber R, Tononi G, Cirelli C (2007). *Drosophila Hyperkinetic *mutants have reduced sleep and impaired memory. J Neurosci.

[B5] Misonou H, Trimmer JS (2004). Determinants of voltage-gated potassium channel surface expression and localization in mammalian neurons. Crit Rev Biochem Mol Biol.

[B6] Yuan LL, Chen X (2006). Diversity of potassium channels in neuronal dendrites. Prog Neurobiol.

[B7] Guan D, Lee JC, Tkatch T, Surmeier DJ, Armstrong WE, Foehring RC (2006). Expression and biophysical properties of Kv1 channels in supragranular neocortical pyramidal neurones. J Physiol.

[B8] Lin Y, Han M, Shimada B, Wang L, Gibler TM, Amarakone A, Awad TA, Stormo GD, Van Gelder RN, Taghert PH (2002). Influence of the period-dependent circadian clock on diurnal, circadian, and aperiodic gene expression in *Drosophila melanogaster *. Proc Natl Acad Sci USA.

[B9] Korngreen A, Sakmann B (2000). Voltage-gated K+ channels in layer 5 neocortical pyramidal neurones from young rats: subtypes and gradients. J Physiol.

[B10] Li Z, Okamoto K, Hayashi Y, Sheng M (2004). The importance of dendritic mitochondria in the morphogenesis and plasticity of spines and synapses. Cell.

[B11] Rhodes KJ, Strassle BW, Monaghan MM, Bekele-Arcuri Z, Matos MF, Trimmer JS (1997). Association and colocalization of the Kvbeta1 and Kvbeta2 beta-subunits with Kv1 alpha-subunits in mammalian brain K+ channel complexes. J Neurosci.

[B12] Sheng M, Tsaur ML, Jan YN, Jan LY (1992). Subcellular segregation of two A-type K+ channel proteins in rat central neurons. Neuron.

[B13] Sheng M, Tsaur ML, Jan YN, Jan LY (1994). Contrasting subcellular localization of the Kv1.2 K+ channel subunit in different neurons of rat brain. J Neurosci.

[B14] Tsaur ML, Sheng M, Lowenstein DH, Jan YN, Jan LY (1992). Differential expression of K+ channel mRNAs in the rat brain and down-regulation in the hippocampus following seizures. Neuron.

[B15] Steriade M (2006). Grouping of brain rhythms in corticothalamic systems. Neuroscience.

[B16] Douglas C, Southard T, Vyazovskiy V, Messing A, Tononi G, Cirelli C (2006). Voltage-dependent potassium channel Kv1.2: effects on sleep and EEG power spectrum of a null Kv1.2 mutation in mice. Sleep.

[B17] Brew HM, Gittelman J, Silverstein RS, Hanks T, Demas V, Robinson L, Robbins C, McKee-Johnson J, Chiu SY, Messing A, Tempel BL (2007). Seizures and reduced lifespan in mice lacking the potassium channel subunit Kv1.2, but hypoexcitability and enlarged Kv1 currents in auditory neurons. J Neurophysiol.

[B18] Smart SL, Lopantsev V, Zhang CL, Robbins CA, Wang H, Chiu SY, Schwartzkroin PA, Messing A, Tempel BL (1998). Deletion of the K(V)1.1 potassium channel causes epilepsy in mice. Neuron.

[B19] McCormack K, Connor JX, Zhou L, Ho LL, Ganetzky B, Chiu SY, Messing A (2002). Genetic analysis of the mammalian K+ channel beta subunit Kvbeta 2 (Kcnab2). J Biol Chem.

[B20] Zhou BY, Ma W, Huang XY (1998). Specific antibodies to the external vestibule of voltage-gated potassium channels block current. J Gen Physiol.

[B21] Douglas C, Vyazovskiy V, Southard T, Faraguna U, Cirelli C, Tononi G (2006). Voltage-dependent potassium channels Kv1.2: effects on sleep and EEG power spectrum of intracortical injections of an anti-kv1.2 antibody. Sleep.

[B22] Daszuta A, Gambarelli F (1985). Early postnatal development of EEG and sleep-waking cycle in two inbred mouse strains. Brain Res.

[B23] Espinosa F, Marks G, Heintz N, Joho RH (2004). Increased motor drive and sleep loss in mice lacking Kv3-type potassium channels. Genes Brain Behav.

[B24] Vyazovskiy VV, Deboer T, Rudy B, Lau D, Borbely AA, Tobler I (2002). Sleep EEG in mice that are deficient in the potassium channel subunit K.v.3.2. Brain Res.

[B25] Bushey D, Huber R, Tononi G, Cirelli C (2006). Mutations in *Hyperkinetic *(*Hk*) reduce sleep in *Drosophila melanogaster*. Sleep.

[B26] Liguori R, Vincent A, Clover L, Avoni P, Plazzi G, Cortelli P, Baruzzi A, Carey T, Gambetti P, Lugaresi E (2001). Morvan's syndrome: peripheral and central nervous system and cardiac involvement with antibodies to voltage-gated potassium channels. Brain.

